# ‘They’re all individuals, none of them are on the same boat’: barriers to weight management in general practice from the rural nurse perspective

**DOI:** 10.1017/S1463423623000439

**Published:** 2023-07-31

**Authors:** Kimberley Norman, Lisette Burrows, Lynne Chepulis, Hilde Mullins, Ross Lawrenson

**Affiliations:** 1 University of Waikato, Hamilton, New Zealand; 2 Waikato District Health Board, Hamilton, New Zealand

**Keywords:** indigenous, inequity, nurse experience, Nursing, obesity healthcare, primary care, weight management

## Abstract

**Aim:**

To explore nurses’ experiences with, and barriers to, obesity healthcare in rural general practice.

**Background:**

Obesity is a significant health risk worldwide, which can lead to many other physical and psychosocial health issues that contribute to a poor quality of life. Primary care is considered the most suitable context to deliver obesity management healthcare across the world, including New Zealand, which reportedly has 34% of all adults (and 51% Indigenous Māori) classed as obese. Nurses in primary care have a significant role in the multidisciplinary team and deliver obesity healthcare in general practice contexts. Yet, there is little focus on the nurse perspective of weight management, specifically in rural areas where medical staff and resources are limited, and obesity rates are high.

**Methods:**

This was a qualitative research design. Semi-structured interviews with 10 rural nurses from indigenous and non-indigenous health providers were analyzed guided by Braun and Clarke (2006) approach to thematic analysis.

**Findings:**

Three themes were identified: limitations of a nurse role; patient-level barriers; and cultural barriers. Nurses reported experiencing significant barriers to delivering effective weight management in their practice due to factors outside the scope of their practice such as patient-level factors, social determinants of health, rural locality restrictions, and limitations to their role. While this study highlights that practice nurses are versatile with an invaluable skill repertoire, it also demonstrates the near impossibility for rural nurses to meet their rural patient’s complex weight management needs, as there are many social determinants of health, sociocultural, and rural locality factors acting as barriers to effective weight management. Nurses experienced a lack of systemic support in the form of time, resources, funding, and effective weight management referral options. Future investigation should look to address the unique rural weight management healthcare needs that experience many barriers.

## Introduction

Obesity is a significant health issue worldwide, with New Zealand (NZ) ranked the third most obese nation in the Organization for Economic Cooperation and Development (OECD) (OECD, 2017) with 34% of NZ adults classed as obese (51% for indigenous Māori and 71% Pacific) (Ministry of Health, 2021a). In recent history, obesity rates have consistently increased, with over 55% of the global rise in obesity reported to be from rural regions (from 1985–2017) (NCD Risk Factor Collaboration, 2019). In NZ, rural communities and indigenous Māori are reported to have higher rates of obesity than their urban and non-Māori counterparts, and those living in the most deprived communities are 1.6 times more likely to be classed as obese (Ministry of Health, 2021a; NCD Risk Factor Collaboration, 2019). The most recent (2002–2003) NZ health survey reported that rural females were more likely to be overweight or obese than urban females, while there was little difference between urban and rural males (Triggs *et al.*, 2007). However, despite there being no updated rural-specific obesity prevalence figure in NZ, it is likely that the obesity rate has increased. As shown in more recent reports, many rural areas in NZ are classified as high-deprivation (2018) (Environmental Health Indicators New Zealand, 2018) and the 2020/2021 NZ health survey highlighted the overall adult obesity rate increased to 34.3% from 31.2% in one year (Ministry of Health, 2022). With over 600,000 people living in rural NZ (Rural Health Alliance Aotearoa New Zealand, 2019), obesity is a health issue (World Health Organization, 2021a), which is putting significant time, resource, and financial strain on the NZ health system. Currently, one of the greatest impacts on rural health is obesity and its related physiological (type 2 diabetes, cardiovascular disease, and some cancers) and psychosocial (anxiety, depression, body dissatisfaction, social isolation, and poor self-esteem) complications (World Health Organization, 2021b; Ministry of Health, 2022). Rural areas notably experience more challenges with accessing primary healthcare than urban areas, with barriers such as rural geographical location, socioeconomic deprivation, transport, telecommunications, and price of healthcare all contributing to the risk of obesity development (Brewis, 2010).

Primary healthcare professionals, specifically nurses in general practice and nurses in Māori health provider clinics, are regarded as best suited to deliver weight management in NZ due to the frequency in which they see their patients over long periods of time (Ministry of Health, 2017). Similar to other countries’ protocols, including Australia, United Kingdom, Canada, and America (Moyer, 2012; National Health and Medical Research Council, 2013; National Institute for Health and Care Excellence (NICE), 2014; Obesity Canada, 2022), NZ nurses have support from the Clinical Guidelines for Weight Management (CGWM) (Ministry of Health, 2017), which outline best processes for delivery of obesity healthcare. This includes processes for monitoring, assessing, managing, and maintaining patient weight as well as advice for weight management strategies that can be referred to through general practice (Ministry of Health, 2017). Weight management strategies available in general practice include evidence-based dietary advice, very low-calorie diets, exercise programs, commercial weight loss groups (such as Weight Watchers), meal replacement programs, telehealth or mobile programs, weight loss medication, or bariatric surgery (Gudzune *et al*., 2015; Hebden *et al.*, 2013; Ministry of Health, 2017; Te Morenga *et al.*, 2018), which is similar to other high-income countries (Moyer, 2012; National Health and Medical Research Council, 2013; NICE, 2014; Obesity Canada, 2022). General practice clinicians are tasked with delivering weight management healthcare, however, with the current healthcare clinician shortage not only in NZ (GP Pulse, 2022) but across the world (Royal College General Practitioners, 2021), nurses are taking on more of a role of supporting and managing patients. Rural general practice nurses are significantly impacted by the decline in GPs, where they end up taking on extra duties to alleviate this gap (Doolan-Noble *et al*., 2019a), so much so that a rural nurse specialist role has been established (Bell *et al*., 2018) to alleviate some of this strain. Nurses have excellent skills in healthcare including health promotion, building strong therapeutic relationships with patients and their families, providing holistic healthcare, public health education, research for informing practice, and understanding their community health needs (Bell *et al*., 2018; McRobbie *et al.*, 2008; Schwerdtle *et al.*, 2020) who are a key general practice healthcare team member. However, obesity is a complex health issue with a myriad of contributing factors, including social determinants of health, psychological health, sociocultural norms, and political climates that shape individual lives (Brewis, 2010; British Psychological Society, 2019; World Health Organization, 2023). Identifying the appropriate weight management strategy that suits a patient is a complicated task for a nurse as one weight management tactic ‘does not fit all’.

Overall, recent literature has indicated that an effective weight management strategy in primary care includes a combination of dietary control, exercise engagement, and behavioral changes actioned in culturally appropriate ways (Norman *et al.*, 2021), however, there are barriers to achieving this. Previous literature has indicated that nurses feel under-equipped to tackle weight management (Croghan *et al.*, 2019), have a perceived lack of expertise in motivating patients, lack access to culturally appropriate resources (Nolan *et al.*, 2012), barriers around discussing obesity (Phillips *et al.*, 2014), lack of time or access to a dietitian (Abbott *et al.*, 2021), and lack of clarity around the nurse role or nurse protocol within their practice (Bell *et al*., 2018; Nolan *et al.*, 2012; Van Dillen & Hiddink, 2014). Yet, these studies were not solely focused on the rural nurse perspective. One Wales-based study, that included urban and rural nurses, found that barriers included not wanting to offend their patients, a range of professional perspectives about how to discuss weight management, and a lack of clear effective messages for positive health changes (Phillips *et al.*, 2014). In NZ, barriers to weight management in general practice have been explored by GP (Claridge *et al.*, 2014; Gray *et al.*, 2018; Norman *et al.*, 2022), pharmacist (Gray *et al.*, 2016), and patient perspectives (Doolan-Noble *et al*., 2019b; Russell & Carryer, 2013; Norman *et al.*, 2022; Norman *et al.*, 2023) with only some of these solely focused on rural experiences. In addition, weight management strategies conducted with a Māori or Pacific cultural worldview outside the general practice context have also identified barriers (Bell *et al.*, 2001; Forrest *et al.*, 2016; Eggleton *et al.*, 2018). Rural communities experience many healthcare inequities, including weight management intervention access (National Health Committee, 2010; Rural Health Alliance Aotearoa New Zealand, 2019; Norman *et al.*, 2022). However, while some of these studies (both western-centric and indigenous) include small numbers of rural participants, none of these studies focus on the rural nurse perspective of weight management despite having a significant role in this healthcare delivery. Overall, there is limited understanding of weight management in rural general practice from the nurse perspective worldwide, in NZ, and even less in the Waikato region, which has a large rural and Māori population (Ministry of Health, 2021b). Obesity and its related comorbidities are putting significant strain on the health system, overloading the workload for an already short-staffed workforce (GP Ministry of Health, 2013, 2022; GP Pulse, 2022; Thomas, 2023) and warrants attention.

## Methods

### Aims

The aim of this study was to explore the rural nurse practitioner experience with barriers to delivering weight management healthcare in their practice with a view to identifying areas of healthcare improvement.

### Design

A case study design was used for this study, focussing on understanding the perspectives of a group of nurses who each worked in rural general practices. Increasingly, qualitative research is being sought and drawn on by policymakers and health professionals as the power of ‘story’ can resonate with, disrupt, or generate deeper understanding about existing knowledge. Qualitative research is useful in understanding the context of the lives that people are endeavoring to live with particular conditions. The nurses’ narrative will resonate with others as they point to the front-line experiences of people trying to do their best work in trying circumstances. The qualitative stories permit an understanding of the challenges but also of the care and skill with which nurses approach their work in obesity management care. As with many qualitative designs, semi-structured interviews were used to elicit the perspectives of participants and as signaled below, content analysis was deployed to draw out themes from the nurses’ narratives (Braun & Clarke, 2006).

### Participants

Practice nurses were recruited through rural general practices throughout the Waikato region. Rural general practices and Māori health providers in rural Waikato were contacted via email with an information sheet and consent form and invited to circulate the invitation to participate to their nurses and contact the researcher (KN) if they would like to take part. Māori health providers were included specifically to ensure indigenous health worldview perspectives of nurses were enabled to be collected and generate comprehensive findings. Inclusion criteria were a registered and currently practicing nurse practitioner from a rural general practice or a rural Māori health provider who delivered weight management in their role. There were no age, gender, ethnicity, or years in their role exclusion criteria. Rural locality was defined as per the Geographical Classification for Health (Whitehead *et al.*, 2021). Once initial contact was made, any questions or concerns responded to, and the participant had agreed to participate, a suitable interview time and location were arranged at the convenience of the participant. Ten rural nurses volunteered to take part in this study from various rural localities. All were female and ranged from three to over twenty years of experience as a practicing nurse.

### Data collection

Semi-structured, open-ended interviews were held between May–October 2021. The researcher (KN) traveled to the participants to avoid potential rural location barriers for participating (Davis-Wheaton, 2013). Face-to-face interviews were conducted in sites including rural general practices, local cafés, and Zoom interviews were held to align with relevant Covid-19 lockdown restrictions. All appropriate cultural considerations were accommodated, including time and space for prayer, karakia (Māori prayer), introductions, or other appropriate meeting opening/closing. All participants were reminded of their rights, the anonymous and confidential nature of the research, and any further questions or concerns were answered by the researcher before informed consent was granted. Once written consent forms were signed and verbal permission to audio record was granted, the interview commenced, and participants were given a $30 voucher as recognition of their time. Participants were reminded they may pause, cease completely, and withdraw from participating at any stage with no questions asked.

Semi-structured interview questions were used to elicit understanding of the nurses’ perspectives. Open-ended questions and the use of an interview guide (developed by whole research team) enabled a broad range of material to be discussed and assisted in assuring participants felt able to lead conversations in directions that mattered to them. Questions included: ‘Please tell me about your experience with delivering weight management healthcare in your practice?’, ‘Could you please tell me about any barriers you have experienced with obesity healthcare in your practice?’, and ‘From your perspective, how effective do you find weight management strategies in your role and general practice?’. Interviews lasted up to 60 minutes, participants were thanked for their time, offered to review transcripts (none did), and the interview was closed. No follow-up interviews were conducted and no participants withdrew from the study.

### Ethical considerations

Ethical approval was granted by the University’s Human Research Ethics Committee.

### Data analysis

Interviews were audio recorded and transcribed verbatim. A deductive approach to analysis of the qualitative material was used, whereby the coding and theme development were framed within the existing concept of ‘barriers’ (Braun & Clarke, 2006). Transcripts were printed out, read, and reread by two researchers (KN and LB). In the left-hand margin, passages of text were highlighted representing any barriers expressed in the interview and labeled with a code. In the right-hand margin, passages of text that were significant to weight management experiences were highlighted and labeled with a code as well, to enable any novel aspects of weight management to be identified from the participant narratives. All codes were listed out, with any double-ups, redundant, or irrelevant codes removed. Three participants’ (from different rural localities) code lists were synthesized to form initial (seven) themes in collaboration with the wider research team (KN, LB, LC, HM, and RL). The research team varied in experience to minimize bias, gain deeper insight, and generate comprehensive findings. This team consisted of emerging, middle, and tenured academic researchers, clinical professionals (rural GP and nurse), and one member with lived experience with obesity and weight loss. All transcripts were revisited to check that these themes were evident in the data by two researchers (KN and LB). COREQ checklist was used for this study. While the ability to achieve data saturation is considered to be situated and subjective (Braun & Clarke, 2021), this analysis continued until the two researchers (KN and LB) agreed that no fresh themes were identifiable in the data. From this, a total of three overarching themes were identified: the limited scope of a nurse, patient-level barriers, and cultural barriers.

## Results

### Limitations of a nurse role

Most nurses regarded comprehensive weight management as something that lay outside of the scope of their role. Most agreed that the effectiveness of any weight management strategy depends on the peculiarities of individual patients’ circumstances and experiences. Many nurses found themselves fulfilling roles such as a health coach, educator, well-being advisor, counselor, facilitator for access to other social services, patient social support, or a motivational healthcare professional, depending on their patients’ individual needs. One nurse described an encounter with a patient that clearly points to the complex nexus of factors present in any delivery of obesity health care. Psychological factors, historical trauma, patterns of eating behavior, and nutritional advice all came into play in the following exchange:

*‘So, we talk about the psychology of- and why they’re perhaps overweight. Talk about the upbringing, how the childhood perhaps had an impact on the weight. Often, they’re-often people with depression, and other mental health problems. We usually go through, get a pen and paper and we write down what they ate on a daily basis. Generally, we talk about how often they have takeaways, and the impact of that, and the impact of obesity on the health and the future, what the future is going to be like being obese, as they grow older. Yeah that’s probably the gist of it. We sort out a meal plan. We talk about how different foods impact on the body, carbohydrates and sugars and fat’ (Nurse 03)*

As several of the nurses pointed out, they are not necessarily trained to address obesity management in these kinds of ways, nor is there time in a regular nurse/patient appointment to engage in these kinds of ‘counseling’ practices. As one nurse put it:

*‘It seems like the more effective programs I’ve seen lately are- use more* counselling *as to why you’re eating that way or motivational therapy, and we just don’t have the training or the time to do that at the moment’ (Nurse 01)*

Several acknowledged that discussing obesity with clients is delicate and challenging at the best of times, but even more so within the short time frame allocated for appointments. As one nurse put it, *‘… you won’t even touch on weight until you’ve got a therapeutic relationship that we can even talk about it!’ (Nurse 02).*

Furthermore, given the multifaceted nature of obesity, many nurses were highly attuned to the need for a holistic approach to address the range of factors involved. The constraints of the current model of care they operated in were often acknowledged as barriers to approaching health improvement in this holistic fashion. In other words, the systems they worked within were not necessarily set up to deal with the complexities of individual needs/experiences. As one nurse put it:

*‘So, yes, definitely, you have to have a well suited model of care in the practice that encompasses a holistic health journey for people, otherwise, obesity [management] will never happen. Sometimes obesity is the last thing they’re worried about, they’re worried about the thing that is happening right now. The cold, the flu, the broken arm from rugby practice. And the obesity is the elephant in the room because it’s not talked about. They don’t want to talk about it. Because our general practice model is set up for 15 minute consults- there is no time’ (Nurse 02)*

Many nurses stressed that often patients had other health concerns (physical or psychosocial) that were more important for them than weight specifically. This indicated the intricate nature of obesity and how the role of a nurse is applied to real-world contexts beyond the clinical or nutritional weight management needs. In many cases, weight loss was positioned as a by-product of adopting a healthier lifestyle, which enabled overall health improvements for patients. As stressed by one nurse, simply telling someone to lose weight was not an effective strategy for achieving patient health improvement, and instead utilizing a patient-centered approach aligned with their (perhaps nonclinical orientated) goals was more effective:

*‘[I’d say] but what are their goals? Your [practitioner] goals are to lower blood sugar, it might not be theirs [patients]. Their goal might be to live to see their grandchild. Their goal might be to walk to the letter box without having to stop. Until they can reach their goal, any goals you have as health professionals- we actually [have to be] really careful we don’t project our own on them. Whilst they might be genuine and good for society. I’m sorry if it’s not their goals then it’s not gonna happen. They might not know that within seven days of walking to the letter box it might be really surprising that they lose weight. It’s important that we are making sure that we’re going and striving for change with them’ (Nurse 02)*

More training or referral pathways were reportedly needed for effective weight management healthcare given the minimal time available in primary care. Nurses expressed a desire to provide help to their patients in ways that suited them, yet they were limited in what they could offer patients. As detailed by one nurse:

*‘More training around different strategies would be great, or programs around- and even the counselling side of it, being more able to give strategies, I know, some mindfulness stuff which… I know that’s been proven to be helpful for a lot of people, but just kind of more strategies. Other than [shake diet] and go with more education, and then more programs that are easier for us to just sign our patients up to, because it’s hard for us to find the time’ (Nurse 01)*

Nurses reported that delivering weight management in their practice was complex and required many different skills to be effective and tailored for each patient. They identified education, scope of practice, time, and system limitations in their current roles.

### Patient barriers

Nurses largely understood that there were numerous patient-level barriers to effective weight management healthcare delivery, including an obesogenic environment, the presence of social determinants of health, psychosocial issues, and life stressors. Many nurses indicated that before even addressing nutritional components of weight management, other factors required urgent attention in the short time frame available. As one nurse described:

*‘Like I say it’s addressing the underlying issues. If they are stressed out with their finances, they are not going to be in the right head space to want to go be thinking ‘I need to exercise or eat healthy’. You’ve got to address the underlying issues [like] depression or family violence- whatever. You’ve got to do the wrap around to get your outcome. It’s no use just addressing one thing and just putting your finger in a hole- it’s going to blow somewhere else. For sure doing a proper assessment [is important but] then that comes down to time. You’ve got 10 minute appointments, often triple booked. And if you’ve got the time to spend with them to get to know them, engagement there, then you can often link in with other services’ (Nurse 09)*

Most of the nurses found that patient-level factors were challenging to address as nurses. Often, referral to financial or social services was needed as many rural patients were living in high-deprivation communities with unhealthy food environments where ‘healthy eating’ was considered to be out of financial reach. One nurse highlighted how important it is to understand the patient-level factors impacting their health to be able to offer relevant advice:

*‘You need to get into their mindset of where they’re at in life, what they’re doing, what they can afford, what they can’t afford’ (Nurse 04)*

Referral to other tailored health services were also reported to be out of financial reach for many of their patients in need:

*‘Then it’s expensive if you refer someone to anything- so a dietician is expensive, personal trainers are really expensive. Like, all of those things are expensive, and people just don’t have access to them’ (Nurse 10)*

Instead, nurses offered ‘practical advice’ for patients to improve health outcomes in ways that were regarded as feasible. This included strategies such as removing all high-sugar soda drinks from the home and swapping it for water, or avoiding snacking on high fat, high salt, high sugar, or foods. As one nurse expressed:

‘*Cutting down from dark blue milk to light blue, getting rid of your cream… Or if you’re getting takeouts, don’t go to a KFC, there are better options. So [we] don’t say ‘do not eat’, you ‘should not’ have. [Instead] offer them options that you know are going to work*’ (Nurse 09)

Or alternative exercise options that are feasible, as described by one nurse:

*‘You don’t have to go to a gym, just walk. Walk for an hour a day if you can, play with your kids more, rather than sitting on the couch*’ (Nurse 09)

Most of the nurses stressed a need to ensure any advice given in their practice took into consideration the unique patient-level factors that can be hindering weight management efforts. Offering practical food and exercise advice was identified to be best practice in their role for patient well-being.

### Cultural barriers

Many nurses identified that the available weight management options in primary care were not necessarily realistic, nor accessible, for their rural or indigenous patients. Poverty, lack of access to public transport or private car, and mobility issues were just a few of the barriers to engagement with programs. As one nurse described:

‘*You know, knee problems, hip problems, problems with obesity are huge. So if you want to say, look, we need to look at low impact exercise, like swimming or cycling. One, they don’t have access to a pool. And if they do, they have to travel and you know, it’s not always optional*’ (Nurse 09)

To counteract this inaccessibility, nurses attempted to provide practical and feasible options for exercise. As expressed by one nurse:

‘*Trying to show them basic exercises that they can do at home [helps] if they don’t want to go walk the streets, they could walk around their house X amount of times, if they’ve got stairs they can go up and down steps*’ (Nurse 04)

Many nurses highlighted that the available weight management strategies were not tailored for the wide range of cultures that make up NZ unique population, which acted as a barrier in their practice. One nurse highlighted the intersection of barriers that existed for some patients, whereby financial affordability, access to transport, cultural food practices, and rural lifestyle limitations rendered offering and following recommended nutritional guidelines difficult:

‘*They can’t afford it! Yeah when I worked for [health practice] they wanted me to do a kete, like the [food] pyramid and I was in [inland town] and they wanted me to go and get fresh fish, and kina, and I’m thinking- where the hell am I going to get that? Yes we are Māori- but it’s only if someone is going up the coast that’s got a boat- which no one’s really gotta boat- and what about the rural people that are stuck way out? They are not gonna go and think I’ll have salmon and salad for dinner*’ (Nurse 05)

Some nurses stressed that while there were some resources available about best practice nutritional guidelines, these were not always relevant to their rural patients’ lifestyles. Instead, nurses attempted to offer culturally relevant advice, however, this was expressed with a sense of lack of confidence as there was no standardized nutritional information to offer in their practice. As one nurse indicated:

‘*We have a lot of Samoans and Indians, so you can tailor it a little bit, you know, what rice do you use? What oil do you use? I don’t have a huge knowledge of other diets or other ethnicities, but it’s a starting poin*t’ (Nurse 09)

Overall, nurses reported to have many facets to their role with weight management, which was difficult to deliver due to patient-level barriers, rural or cultural lifestyles, and lack of access to programs that are relevant or feasible for patients in their rural practice.

## Discussion

Analysis across the three themes discussed above would suggest that nurses’ role in weight management is a multilayered and complex affair. Patient-level factors (such as social determinants of health) necessarily became part of a nurse’s role when delivering weight management care. Nurses found themselves facilitating access to social services, behaving like a counselor and health coach, and operating as pedagogues, tailoring ‘education’ to different patients’ needs. Their role was a ‘holistic’ one, yet current models of care were not necessarily set up to permit this holistic approach. In many cases, before even offering a nutritional plan for physiological weight loss, nurses needed to address other aspects of their patient’s health needs, such as ensuring a safe environment, firming up financial status, and assessing psychological or sociocultural situations.

The role described above is a multifaceted one, yet significant time, resource, and support restrictions constrained nurses’ capacity to deliver quality obesity health care in the ways they would have liked to. Nurses are not formally trained to be counselors, motivational therapists, dietitians, educators, or behavioral change psychologists, yet they needed to be one or all of these things to support and empower their patients. Many nurses called for more time in their role to up-skill their training and education in the weight management field. They wanted to enhance their capacity to meet the diverse patient needs in this area.

Effective obesity healthcare has been identified to utilize a multidisciplinary approach (Anderson *et al.*, 2021; Bischoff *et al*., 2017), including qualified pharmacist prescribers, dietitians, psychologists, and social care workers each of whom have years of training and development around the intricacies of weight management. However, these teams are rarely present or accessible on a consistent basis for rural general practices in NZ, which is potentially perpetuating the heavy and expanded workload that falls into nurses’ laps. While this study offers support for previous literature that indicated the role of a rural practice nurse in weight management is undefined, with different views of a nurses’ professional responsibilities and boundaries (Bell *et al*., 2018; Doolan-Noble *et al*., 2019a), this study sheds light on the extent and range of extra responsibilities rural nurses take on in a real-world context. It was encouraging to find that rural nurses go above and beyond their job description. However, given the already reported time, funding, staffing, training, and resource constraints of rural health experiences (Alsop-ten Hove, 2019; Davis-Wheaton, 2013; National Health Committee, 2010), it may be unreasonable to expect rural nurses to provide services they are not specialists in for such a complex and unique health issue. Instead of further overloading the already strained role of a nurse (Bennett *et al.*, 2012; Doolan-Noble *et al*., 2019a), this study recommends that rural nurses be systemically supported with access to a wider multidisciplinary team, ideally based on general practice, to offer the range of weight management services that rural communities reportedly could benefit from.

Rural locality was positioned as a barrier hindering many weight management efforts. Nurses stressed that many of the options available through general practice (or outlined in the CGWM) (Ministry of Health, 2017) were predominantly ‘impractical’ or ‘unsuitable’ for their rural patients who were living in high-deprivation areas with no financial means or transport to attend programs or exercise facilities located out of town. While it is recommended that any weight management healthcare be delivered in culturally appropriate ways (CGWM), these nurses indicated a lack of resources or access to information that is specific to the range of cultural food norms practiced in NZ, including those of Māori. While these findings align with previous rural patient (Norman *et al.*, 2022; 2023) and GP perspective literature that highlights the complexities of effective weight management in rural settings (Norman *et al.*, 2022), this study extends this to the often overlooked rural nurse experience. Nurses offered patients ‘practical’ or ‘realistic’ obesity-related health advice (such as changing to low-fat milk, removing high-sugar drinks out of the house, and home exercises) – advice that they felt patients could conceivably achieve. While not explicitly labeled as such by the participants, this ‘practical’ advice and behavior in their practice would indicate that these nurses’ care aligned with the ethos of contemporary movements such as ‘Health at any Size’ (Bacon, 2010; Bacon & Aphramor, 2011). Focussing less on a (notably flawed) BMI scale (Bhurosy & Jeewon, 2013) or weight number and more on health-related improvements (such as lowering risk of stroke, heart disease, or diabetes through small changes to diet and exercise in sustainable formats) was stressed as ‘best practice’ for these rural nurses given the limited ‘suitable’ weight management referral options to offer patients. It is commendable that rural nurses are providing beneficial healthcare in areas experiencing significant accessibility issues (National Health Committee, 2010) by tailoring health advice to meet the sociocultural norms and socioeconomic limitations of communities (Coupe *et al.*, 2018; Verbiest *et al*., 2018). However, it also sheds light on the difficulties experienced and near impossibility for rural nurses to meet their rural patients’ complex weight management needs, as there are many social determinants of health and environmental factors affecting their rural patients (Swinburn *et al.*, 1999; World Health Organization, 2023) that are outside the scope of a nurse or general practice control.

### Implications and future directions

This study sheds light on an important member of rural health team, the nurse, which has been given little attention, despite having a significant role in weight management healthcare in general practice. Overall, this study highlighted a myriad of extra responsibilities a rural nurse actions within a more restrictive health access climate when compared to their urban counterparts and warrants further attention. Further investigation into how to better support rural nurses working in the weight management general practice context in high-deprivation communities is recommended. NZ is currently undergoing significant health reforms, with rural health being recognized as a unique and stand-alone health sector moving forward due to the different health needs than urban (Rural Health Alliance Aotearoa New Zealand, 2019; New Zealand Government, 2022). In addition, clinician burnout from heavy workloads and significant staff shortages across NZ are adding to the already reported largely autonomous, complex, and heavy workload of a rural nurse (Doolan-Noble *et al*., 2019a; Goodyear-Smith & Janes, 2008). Difficulties in retention and professional development for rural nurses have been indicated in previous NZ literature (Carryer *et al.*, 2011; Doolan-Noble *et al*., 2019a). Further to this, recent studies have indicated the gap in systemic support for rural health in areas other than obesity, including the Covid-19 prevention and vaccination priority (Whitehead *et al.*, 2022). This lack of systemic support for rural healthcare and rural nurses working in weight management would benefit from being addressed if the current rural health workforce is to grow and be maintained long term. Rural general practice, and especially the multifaceted rural nurse role, should be prioritized for funding, patient referral access to multidisciplinary teams, ‘practical’ rural weight management intervention options, and staff training to reduce the strain on the rural nurse, improve working conditions for rural nurses, and assist with providing quality health care to improve rural patient health outcomes.

Future directions should look to explore the key barriers this study found to identify areas for improvement of weight management healthcare services rurally. This includes exploring the social determinants of health impacting rural communities, investigating the lack of ‘practical’ culturally appropriate or tailored weight management resources in rural general practices, and a lack of rurally appropriate weight management referral options. Cultural norms play a significant role in dietary consumption and have been identified as a contributing factor to obesity (Ball *et al.*, 2010), which can also extend into the rural cultural lifestyle. In addition, grounding practice in indigenous health models has been demonstrated to improve health outcomes for indigenous populations and should be focussed on for Māori (Ministry of Health, 2015; Forrest *et al.*, 2016; Campbell *et al.*, 2017; Eggleton *et al.*, 2018). However, potentially, the intersectionality of obesity, culturally specific worldviews, and social determinants of health should be further investigated to include a rural/urban intersection as well as rural health needs are recognized to be different from urban.

### Limitations

As with any qualitative study, the findings cannot be generalized. However, this study aimed to explore the perspectives of rural Waikato nurses, who are already a small homogenous sample to elicit data from and are transferable to other rural general practices across NZ. Due to Covid-19 restrictions, only ten nurses were able to participate. Including more participants with a wider ethnicity, age, and male nurse perspectives could generate more nuances across the narratives. However, data saturation was considered to be reached with no new themes emerging from nurse narratives. While this research did include Māori voices, was guided by a cultural advisor, and utilized two data researchers with a reflexive approach, a Kaupapa Māori methodology could elicit richer Māori data and findings relevant to the Māori population.

## Conclusion

This study found that nurses experience barriers to delivering effective weight management in their practice due to factors outside the scope of their practice such as patient-level factors, social determinants of health, rural locality restrictions, and limitations to their role. Nurses were found to go above and beyond their role description to accommodate the myriad of weight-related needs for their patients, however, they experience a lack of systemic support in the form of time, resources, funding, and effective weight management referral options. Future investigation should look to address the unique rural weight management healthcare needs that experience many barriers. Nurses provide an invaluable contribution to the primary care team, however, more support for rural nurses is required to deliver effective healthcare to rural communities and reduce the workload strain on the rural nurse workforce.
